# The Sequence Structures of Human MicroRNA Molecules and Their Implications

**DOI:** 10.1371/journal.pone.0054215

**Published:** 2013-01-18

**Authors:** Zhide Fang, Ruofei Du, Andrea Edwards, Erik K. Flemington, Kun Zhang

**Affiliations:** 1 Biostatistics Program, School of Public Health, Louisiana State University Health Sciences Center, New Orleans, Louisiana, United States of America; 2 Department of Computer Science, Xavier University of Louisiana, New Orleans, Louisiana, United States of America; 3 Department of Pathology, Tulane University School of Medicine, New Orleans, Louisiana, United States of America; College of Pharmacy, University of Florida, United States of America

## Abstract

The count of the nucleotides in a cloned, short genomic sequence has become an important criterion to annotate such a sequence as a miRNA molecule. While the majority of human mature miRNA sequences consist of 22 nucleotides, there exists discrepancy in the characteristic lengths of the miRNA sequences. There is also a lack of systematic studies on such length distribution and on the biological factors that are related to or may affect this length. In this paper, we intend to fill this gap by investigating the sequence structure of human miRNA molecules using statistics tools. We demonstrate that the traditional discrete probability distributions do not model the length distribution of the human mature miRNAs well, and we obtain the statistical distribution model with a decent fit. We observe that the four nucleotide bases in a miRNA sequence are not randomly distributed, implying that possible structural patterns such as dinucleotide (trinucleotide or higher order) may exist. Furthermore, we study the relationships of this length distribution to multiple important factors such as evolutionary conservation, tumorigenesis, the length of precursor loop structures, and the number of predicted targets. The association between the miRNA sequence length and the distributions of target site counts in corresponding predicted genes is also presented. This study results in several novel findings worthy of further investigation that include: (1) rapid evolution introduces variation to the miRNA sequence length distribution; (2) miRNAs with extreme sequence lengths are unlikely to be cancer-related; and (3) the miRNA sequence length is positively correlated to the precursor length and the number of predicted target genes.

## Introduction

**Figure 1:**
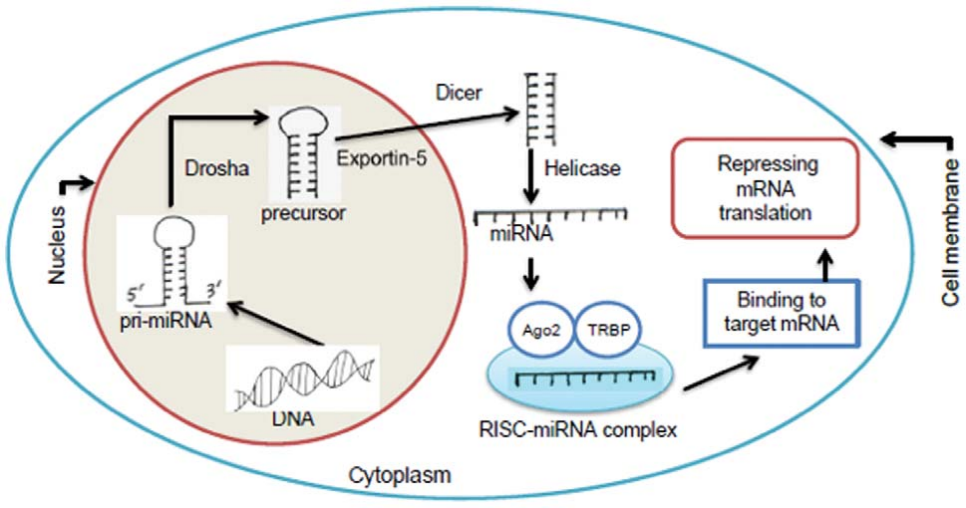
Biogenesis of mature miRNAs and their functional activity.

MicroRNAs (miRNAs) have been identified as a group of small endogenous non-coding RNAs that negatively regulate protein-coding messenger RNAs (mRNAs) at the post-transcriptional level. The derived process and the main activity of a miRNA are clear and well described in the literature. Mature miRNAs are single-stranded RNAs consisting of about 22 nucleotides and are derived from longer non-coding primary miRNAs (pri-miRNAs) and then from precursor miRNAs (pre-miRNAs) by the sequential actions of the Drosha and Dicer RNA cleaving enzymes [1]–[3]. The main function of miRNAs is to step in and intervene in the translation of mRNAs or to induce degradation of the mRNAs. In mammals, mature miRNAs are incorporated into an RNA-inducing silencing complex (RISC). The activated RISC permits the miRNAs to bind to the 3′ untranslated regions (3′UTR) of specific target mRNAs to suppress translation and cause their degradation by mRNA decay [4]–[6]. There may not be a one-to-one correspondence between the miRNAs and targeted mRNAs. A single miRNA may have multiple mRNA targets. It is a challenging task to predict the targeted mRNAs of a miRNA, though the precise prediction is essential to study its functional activity and its association with diseases. The process of deriving a miRNA molecule and its main activity is depicted in **Figure 1**.

To annotate a cloned sequence as a miRNA, the most important criteria include the characteristic length (approximately 22-nucleotide) of the sequence and a corresponding compact pre-miRNA loop structure, with a median of 83 nucleotides [7]. The association between the biological significance and the sequence length heterogeneity has been recently recognized for a mature miRNA in *Arabidopsis thaliana* [8]. This study shows the importance and necessity to study the distributional structure of the sequence lengths of mature miRNA molecules in the genome, and the factors that may affect this length heterogeneity. With the development of profiling technology and the advances of bioinformatics/computational tools, the number of miRNAs identified has increased dramatically. Since the first miRNA was discovered in 1993 [9] and the biological functions of miRNAs were recognized to be conserved in different species in 2000 [10], [11], the number of mature human miRNAs jumped from 152 in August 2004 to 1732 in April 2011, according to miRBase, a database of published miRNA sequences and annotation [12]–[16]. In this paper, we systematically investigate the length distribution of miRNAs, anticipating that the nature of non-uniformity of this distribution can reveal the complexity of the miRNA molecular structures and have implications for genetic research.

## Materials and Methods

### Materials

The sequences of 1732 human mature miRNAs and the corresponding precursor miRNAs were downloaded from the public database miRBase (Release 17, April 2011). All the calculations were carried out using the *R* language.

### Statistical Methods

A random variable is defined to have an asymmetric Laplace distribution if it has density
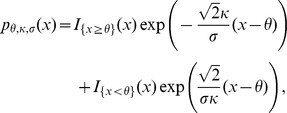
where 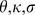 are three unknown parameters. It reduces to symmetric Laplace distribution when 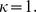 The function 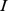 is the indicator function. The maximum likelihood estimates, 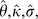 of these parameters are available in [17]. With these estimates, the fitted discrete asymmetric Laplace distribution, DALaplace, has the probability masses defined by,



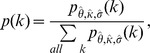
where k ranges from 16 to 27 (the range of the sequence lengths of human mature miRNAs). The discrete symmetric Laplace distribution (DLaplace) is defined in the same fashion.

A zero-inflated Poisson model is defined as
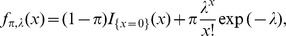
where 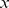 is non-negative integer, and 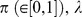 are unknown parameters. This is a mixture model and it reduces to Poisson distribution with mean 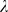 when 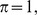 or a single point distribution putting its all mass at zero when 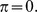 We fitted this model to the absolute differences of mature miRNA sequence lengths and their median, and obtained the maximum likelihood estimates [18]: 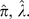 Then the discrete, symmetric zero-inflated model (DSZero-Inf) is defined as,



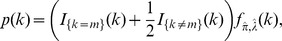
where *k* ranges from 16 to 27, and *m* is the median of the observed sequence lengths of human mature miRNAs.

The tPoisson distribution is defined as
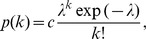
where *k* ranges from 16 to 27, λ is the average sequence length of all human mature miRNAs, and *c* is a constant such that the sum of all probabilities is one.

## Results and Discussion

### The Distribution of the Sizes of Human Mature miRNA Molecules

**Figure 2:**
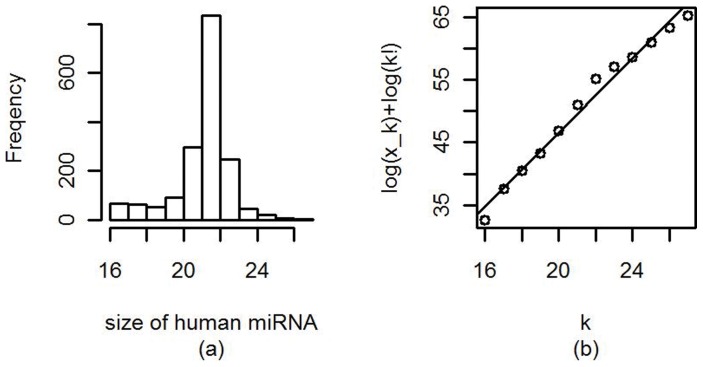
Histogram and corresponding Poissonness plot of the sequence lengths (sizes) of human mature miRNA molecules.

The number of nucleotides in a human mature miRNA is a discrete random variable, which ranges from 16 to 27 and has a mode and a median of 22. A histogram of lengths of all human mature miRNA molecules is presented in **Figure 2**(a). Though the Poisson distribution is the traditional model for fitting the count data, it does not fit the length distribution of mature miRNA molecules well. **Figure 2**(b) is the Poissonness plot of the data [19]. It is created by plotting 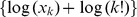 against 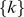, where 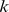 is the count, 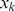 is the corresponding observed frequency and *k!* represents the factorial of *k*. It is clear that the plotted points do not fall onto a straight line, with the points at the middle above the line and the points at both ends below the line. This suggests non-Poisson distribution should be employed to fit the length distribution of mature miRNAs.

A unique feature of Poisson distribution is the equality of its mean and variance. This is not the case for the lengths of human mature miRNA molecules because the sample mean (21.52) is much larger than the sample variance (2.51). This fact also implies that negative binomial distribution, another popular distribution to model the count data and handle the over-dispersion problem in counts, could not fit the human mature miRNA lengths well.

**Figure 3:**
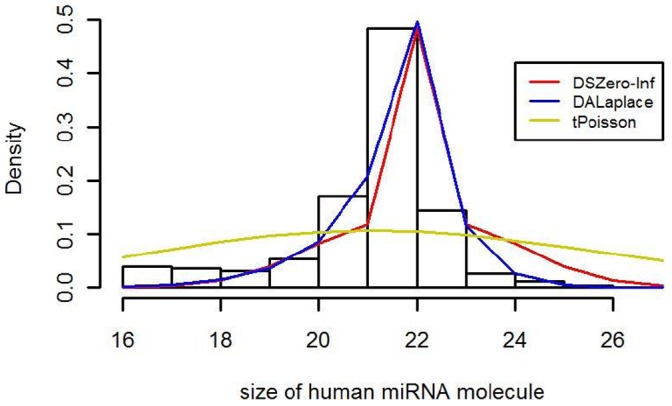
Histogram of sequence lengths of human mature miRNA molecules and four fitted models.

We show in **Figure 3** the schematic fitting results of three discrete distributions to the sequence lengths of human mature miRNA molecules. These include a discrete analogue of the asymmetric Laplace distribution (denoted as DALaplace), a discrete, symmetric distribution induced from the zero-inflated model (denoted as DSZero-Inf) and a truncated Poisson distribution (denoted as tPoisson). Details of DALaplace, DSZero-inf and tPoisson are discussed in the Materials and Methods. Interested readers are referred to [17] for the definition of the asymmetric Laplace distribution and methods for parameter estimation, and to [19] for the definition and applications of the zero-inflated model. It is clear from the plot that DALaplace performs best while tPoisson is the worst. The sum of squares of the residuals (differences between observed percentages and corresponding fitted values) are 0.0047, 0.01, 0.175 for DALaplace, DSZero-inf and tPoisson, respectively, further illustrating the performance of these models. We also calculated AIC (Akaike information criterion) to evaluate the relative goodness of fit of these non-nested models. AICs for DALaplace, DSZero-inf and tPoisson are 5893.396, 6117.659, and 7970.977 respectively. The order of these values confirms our selection of the model.

### The Randomness of Bases in Mature Human miRNA Molecules

**Figure 4:**
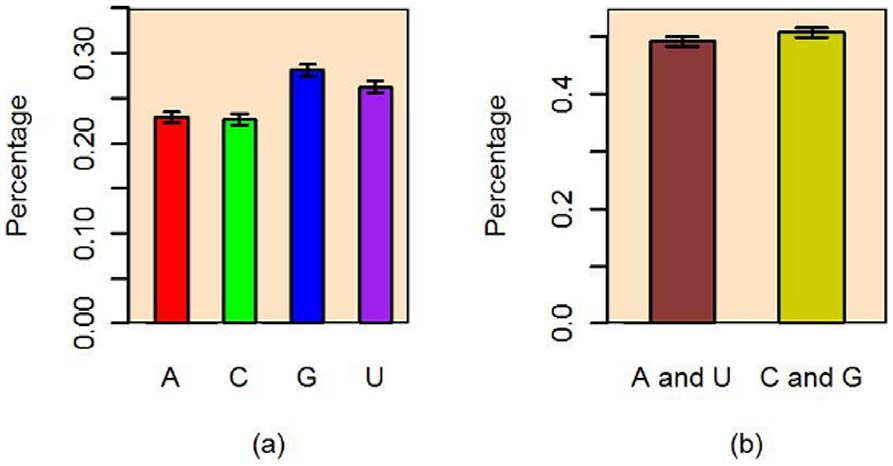
The sample proportions of nucleotide bases and GC, AU contents.

Another question of interest to biologists is whether there is any structural pattern in a human mature miRNA; in other words, whether the proportion of one nucleotide base is significantly higher or lower than those of other bases. We intend to address this problem in this subsection. Given the length of a mature miRNA sequence, the vector of counts, 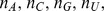
of the bases, *A, C, G, U*, follows a multinomial distribution. By the likelihood ratio method for the test of proportional homogeneity, we conclude that the proportions of the four bases in every sequence are significantly different (p-value ≈ 0). We further find that at the significance level of 0.05, that there are 341 (about 20%) mature miRNA sequences showing inequality of base probabilities. The sample proportions of four bases in all miRNA sequences are presented in **Figure 4**(a), with the 95% simultaneous confidence interval [20] at the top of corresponding bar. These intervals clearly indicate that the four bases are not equally probable in all the sequences. All these findings imply that there may exist structural patterns in the sequences of certain mature miRNAs.

However, as demonstrated in **Figure 4**(b), the content of GC (50.8%) is very close to that of AU (49.2%). The 99.9% confidence interval for the GC content is (0.499, 0.516), which is narrow and covers 0.5. We comment that the hypothesis of the GC content being 50% holds as long as the significance level is set to be greater than 0.0028. This is due to the facts that the sample size N (the total number of bases in all mature miRNA sequences) is large and that in the hypothesis testing of a proportion, the significant probability goes to zero as N approaches infinity.

### The Relationship to Evolutionary Conservation

Highly conserved DNA sequences are thought to have functional value. The genetic conservation across evolution has been an important benchmark for detecting functionally important nucleic acid sequences, and for studying gene interactions in a group of co-regulated genes [21]–[24]. Hirsh and Fraser [25] revealed a negative and highly significant relationship between the importance of a gene and the evolutionary rate. Similar relationship for miRNAs was also studied in the literature. Zhang et al. [26] reported the rapid evolution of some miRNA clusters. In this subsection, we present our findings on the correlation between evolutionary conservation and the length of mature miRNA molecules. To our knowledge, this is the first study exploring this relationship.

**Figure 5:**
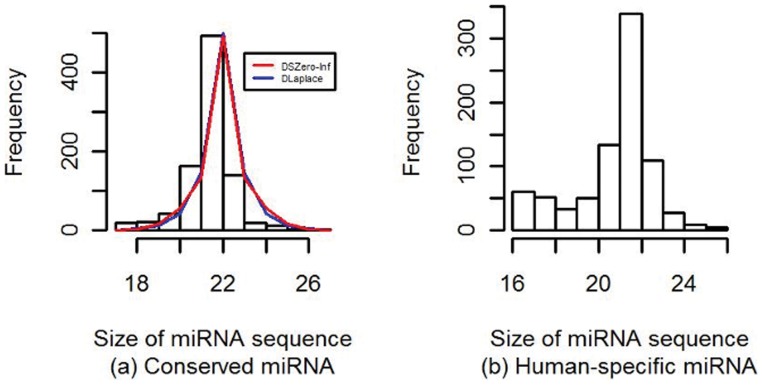
Histograms and fitted distributions of the sequence lengths of mature conserved and human – specific miRNAs.

All human mature miRNAs are divided into two classes, conserved and human-specific, by using the procedure documented in [27]. Out of 1732 mature miRNAs, there are 914 (about 52.8%) miRNAs labeled as conserved and 818 (about 47.2%) as human-specific. These two ratios are significantly different (one-sided p-value is 0.01). **Figure 5** shows the length distributions of the sequences in these two groups. We can see that the sequence lengths of conserved miRNAs are symmetrically distributed around 22. Both the discrete, symmetric, zero-inflated distribution (DSZero-inf) and the discrete, symmetric Laplace (DLaplace) can model the distribution decently and there is little difference between these two models. On the contrary, the sequence lengths of human-specific miRNAs seem to be bi-modally distributed with modes of 16 and 22. One may need a mixture of two distributions to model this variable well. The percentage (7.3%) of the short human-specific miRNAs that have length of 16 or 17 is about ten-fold of that (0.77%) of the short conserved miRNAs (a *Z*-test for equality of two percentages gives a p-value close to 0).

All these results indicate that rapid evolution seems to increase the variation in the sequence lengths of human mature miRNA molecules, and thus complicate the distribution of the length variable.

### The Characteristic Size of Human miRNA Oncogenes and Tumor Suppressors

It has become evident that miRNAs control the expression levels of gene products that are important in cancer progression. A number of studies have shown that many miRNAs reside within chromosomal fragile sites in the human genome and that many miRNAs have been linked to the initiation, progression, and metastasis of human malignancies, with the earlier reports associating miRNAs with cancers being published in [28], [29]. Some miRNAs are able to target oncogenes – those with capacity to induce tumor migration and invasion, or tumor suppressor genes – those with capacity to suppress cancer and metastasis [30]–[33]. The essence of the miRNA’s regulatory mechanism in cancer lies in that increased expression of certain miRNAs can result in down-regulation of tumor suppressor genes, while decreased expression of other miRNAs can lead to increased expression of oncogenes. Examples include hsa-miR-10B [34] and hsa-miR-21 [35] in breast cancer, and hsa-miR-155 [36] in human B cell lymphomas as oncogenes; and hsa-let-7 [37] in lung cancer, and hsa-miR-15 and hsa-miR-16 [28] in chronic lymphocytic leukemia as tumor suppressors.

To investigate the distributions of the sequence lengths of the mature miRNA molecules that are associated with cancer, we generate a class of miRNAs regulating either oncogenes or tumor suppressor genes. For a miRNA to be included, there must be at least one publication indicating the causal relationship between the miRNA and the related oncogene or tumor suppressor gene. We include those miRNAs that play opposite roles in different cancers due to the fact that one miRNA may regulate multiple targets, and the same miRNA may play opposite roles in cancer progression in that it acts as a tumor suppressor in certain cancers and as an oncogene in others [38]. This makes our selection slightly different from that in [16]. If no such a causal relationship exists, a miRNA is selected as an oncogene if it is up-regulated in at least three publications, or as a tumor suppressor if it is down-regulated in at least another three papers. We exclude the miRNAs which show conflicted roles in the same cancer. We obtained 173 cancer related miRNAs listed in **Table 1**, where the function of a miRNA is marked “mixed” if it regulates some oncogenes in a certain cancers and other tumor suppressor genes in different types of cancers. We find that the characteristic sequence lengths of these miRNAs are very stable, with 60.7% of human miRNAs having sequences of 22 nucleotides, 96.5% of human miRNAs having sequences of 22
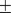
1 nucleotides, and 99.4% of human miRNAs having sequences of 22
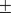
2 nucleotides. The only miRNA whose sequence is of 18 nucleotides, outside of the interval 22
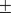
2, is has-miR-516a-3p. This miRNA has connection to human breast cancer progression [39]. The length distribution for the miRNAs exclusively regulating oncogenes (or tumor suppressors) is very similar to that of all cancer-related miRNAs. These observations suggest that an extremely long or short miRNA is unlikely cancer-related. But for a cancer-related miRNA, the sequence length does not affect its classification to oncogenes or tumor suppressors.

**Table 1:** All human mature miRNAs associated with cancer and their functions.

miRNA	Function	miRNA	function	miRNA	Function	miRNA	Function
let-7a	supp	miR-148b	supp	miR-21	onco	miR-34b	supp
let-7a-2*	supp	miR-150	mixed	miR-210	mixed	miR-34c-5p	supp
let-7b	supp	miR-152	supp	miR-214	supp	miR-370	supp
let-7c	supp	miR-153	supp	miR-215	supp	miR-372	onco
let-7d	supp	miR-155	onco	miR-216b	supp	miR-373*	onco
let-7e	supp	miR-15a	supp	miR-218	supp	miR-373	onco
let-7f	supp	miR-15b	supp	miR-219-1-3p	onco	miR-374a	onco
let-7f-1*	onco	miR-16	mixed	miR-22	supp	miR-375	mixed
let-7g	supp	miR-16-1*	mixed	miR-221	onco	miR-376a	supp
let-7i	supp	miR-17	onco	miR-222	onco	miR-376b	supp
miR-1	supp	miR-181a	mixed	miR-223	mixed	miR-377	supp
miR-100	supp	miR-181a-2*	onco	miR-224	onco	miR-424	supp
miR-101	supp	miR-181b	supp	miR-23a	mixed	miR-429	supp
miR-106a	onco	miR-181c	supp	miR-23b	supp	miR-432	supp
miR-106b	onco	miR-182	onco	miR-24-1*	onco	miR-449a	supp
miR-107	mixed	miR-182*	onco	miR-24	onco	miR-451	supp
miR-10a	onco	miR-183	supp	miR-24-2*	onco	miR-485-5p	supp
miR-10b	onco	miR-184	onco	miR-25	onco	miR-486-5p	supp
miR-122	supp	miR-185	supp	miR-26a	mixed	miR-494	onco
miR-124	supp	miR-18a	onco	miR-26b	mixed	miR-495	supp
miR-125a-5p	supp	miR-18a*	supp	miR-27a	onco	miR-497	supp
miR-125b	mixed	miR-191	onco	miR-27b	supp	miR-498	onco
miR-125b-1*	supp	miR-192	supp	miR-296-5p	onco	miR-503	onco
miR-125b-2*	supp	miR-193a-3p	supp	miR-29a	supp	miR-510	onco
miR-126*	mixed	miR-193b	supp	miR-29b	supp	miR-516a-3p	onco
miR-126	mixed	miR-194	supp	miR-29b-2*	supp	miR-519a	supp
miR-127-3p	supp	miR-195	supp	miR-29c	supp	miR-520c-3p	onco
miR-128	supp	miR-196a	mixed	miR-30a	mixed	miR-520h	supp
miR-129-5p	supp	miR-196a*	onco	miR-30a*	supp	miR-521	onco
miR-130b	onco	miR-197	onco	miR-30e	supp	miR-532-5p	onco
miR-133a	supp	miR-199b-5p	supp	miR-31	supp	miR-661	supp
miR-133b	supp	miR-19a	onco	miR-32	onco	miR-675	onco
miR-134	supp	miR-19b	onco	miR-320a	supp	miR-7	supp
miR-135a	supp	miR-19b-2*	onco	miR-324-5p	supp	miR-9	mixed
miR-137	mixed	miR-200a	supp	miR-326	supp	miR-9*	onco
miR-138	supp	miR-200b	supp	miR-328	supp	miR-92a	onco
miR-139-3p	supp	miR-200c	mixed	miR-330-3p	supp	miR-93	onco
miR-140-5	supp	miR-203	supp	miR-335	supp	miR-95	supp
miR-141	supp	miR-204	mixed	miR-337-3p	supp	miR-96	onco
miR-143	mixed	miR-205	supp	miR-340	onco	miR-98	onco
miR-145	supp	miR-206	supp	miR-342-5p	supp	miR-99a	supp
miR-146a	mixed	miR-20a	mixed	miR-345	onco		
miR-146b-5p	supp	miR-20a*	onco	miR-346	onco		
miR-148a	supp	miR-20b	onco	miR-34a	supp		

### The Relationship to the Size of the pre-miRNA

**Figure 6:**
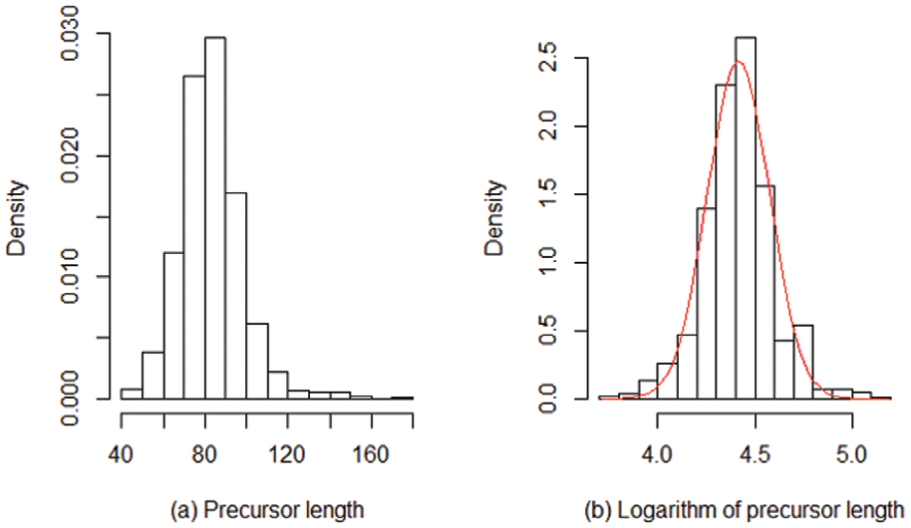
Distributions of the sequence lengths of human pre-miRNAs.

The stem-loop structure of the precursor miRNA is developed prior to the corresponding mature miRNAs. Thus, the association between the biogenesis of a miRNA gene and the sequence features of its stem-loop precursor is also important. Firstly, we study the distribution of the sequence lengths of pre-miRNAs. As presented in **Figure 6**, this distribution has a median (and mode) of 83 nucleotides, but it is skewed to the right (**Figure 6**(a)). A normal model with mean 4.41 (*log*(83)) and standard deviation 0.16 fits the logarithm of the sequence lengths very well (the red curve in **Figure 6**(b) is the fitted model). This indicates that a log-normal distribution can be employed to model the length distribution of the human pre-miRNAs. A good feature is that the log-normal distribution maximizes the entropy probability among distributions whose logarithms have fixed mean and variance [40].

**Figure 7:**
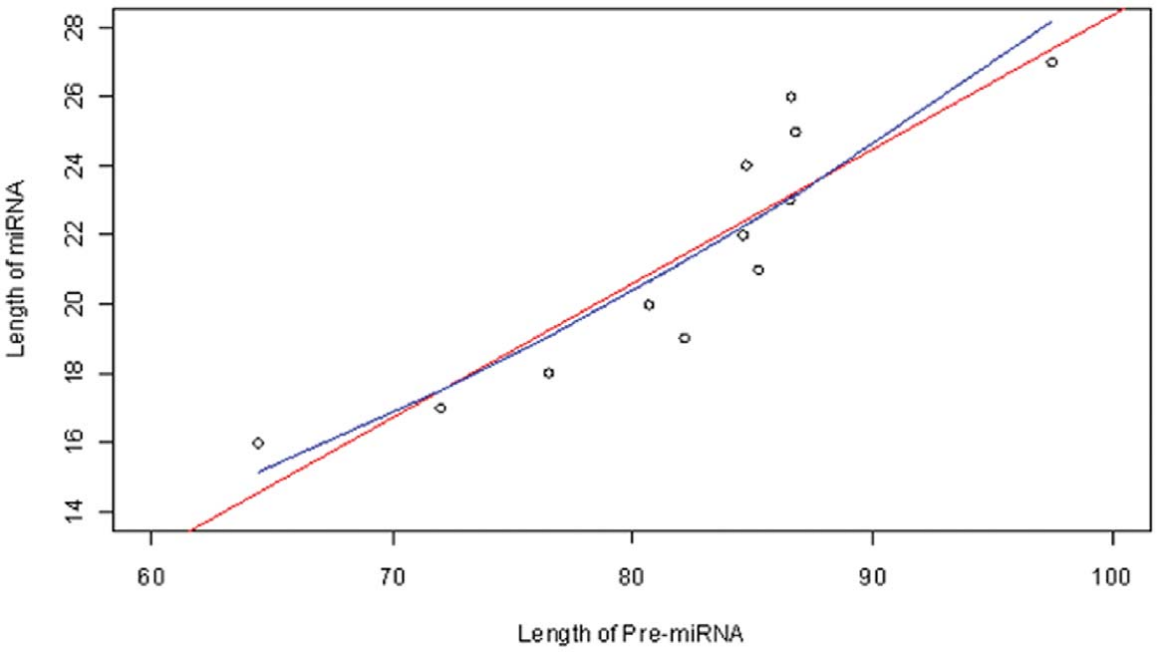
Scatter-plot of the average sequence length of pre-miRNAs versus the sequence length of miRNAs: the red line is the linear regression and the blue curve is the quadratic polynomial regression.

Next we study the relationship between the sequence lengths of human pre-miRNAs and the corresponding mature miRNAs. The sequence length of pre-miRNAs varies from 41 to 180 and there are multiple pre-miRNA lengths corresponding to each mature miRNA length. We first calculate the average sequence lengths of the precursors corresponding to the same mature miRNA length, and then fit the regression model of the mature miRNA length to the average precursor length. As shown in **Figure 7**, there is a positive, significant relationship between the human mature and precursor miRNA sequence lengths (the slope of the red line is 0.388, with p value of 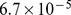). The multiple R^2^ of the regression is 81%. The improvement due to the quadratic polynomial model fit (the blue curve) is not significant, with the p value of the quadratic term equal to 0.539. We also obtained the maximal information coefficient (MIC) and related statistical indexes proposed by Reshef et al. [41]: MIC = 0.65, MIC-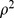 (a measure of non-linearity, 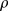 is the Pearson correlation coefficient) = −0.15, MAS (the maximum asymmetry score for non - monotonicity) = 0 and MCN (minimum cell number for complexity) = 2. By comparing these indexes with those in Table S1 in [41], we can conclude a linear association between these two variables – the sequence lengths of human precursor miRNA and mature miRNA.

**Figure 8:**
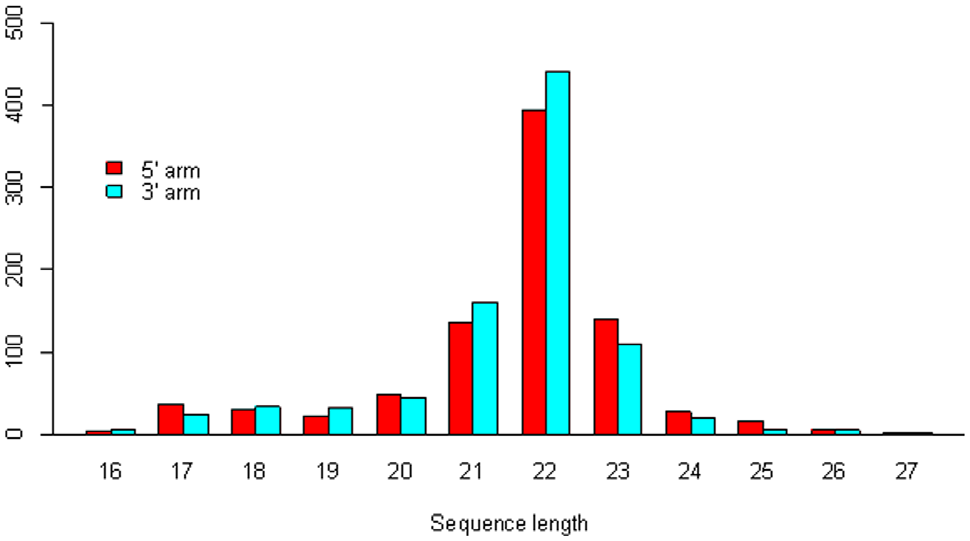
Bar charts of mature miRNAs in the 5′ and 3′ arms of the precursors respectively.

Lastly, we look into where the human mature miRNA resides within its stem-loop precursor – in the 5′ arm or 3′ arm. We scrutinize 1732 mature miRNAs in total, with *has-miR-378d* excluded from the analysis because it locates in both the 3′ arm of the stem-loop precursor *hsa-mir-378d-1* and the 5′ arm of the stem-loop precursor *hsa-mir-378d-2*. There is no significant difference between the percentages of miRNAs in the 5′ arm (49.4%) and 3′ arm (50.6%), and both sequence length distributions are symmetric around approximately 22 (nucleotides). However, as **Figure 8** indicates, difference exists between the sequence length distributions of miRNAs resided in the two arms of the precursors. Longer mature miRNAs (with more than 22 nucleotides) more often locate in the 5′ arm than in the 3′ arm of the precursors. Among the miRNAs located in the 5′ arm and 3′ arm separately, the percentages of the miRNAs with exactly 22 nucleotides are significantly different (46.2% (in the 5′ arm) versus 50.3% (in the 3′ arm), with p value being 0.044).

### The Association of miRNA Sequence Length and the Number of Predicted Target Genes

**Figure 9:**
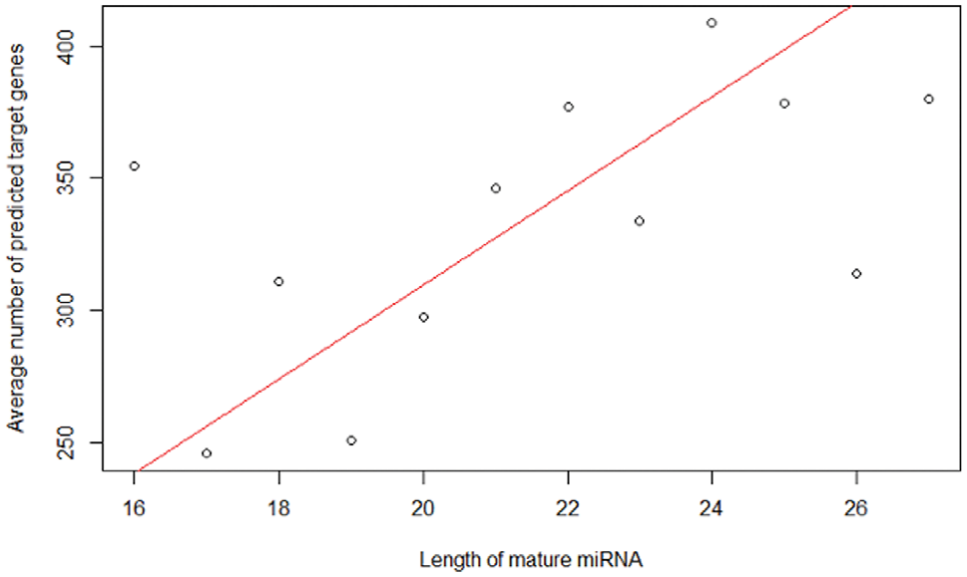
Scatter-plot of the average numbers of predicted target genes versus the mature miRNA sequence length.

A miRNA is a non-coding, functional RNA molecule. Its role in post-transcriptional gene regulation is carried out by binding to the target mRNA and then destabilizing the mRNA or suppressing its translation. In most cases, one miRNA does not bind to a single mRNA but instead binds to multiple targets. The number of predicted target genes varies dramatically from one miRNA to another. Whether this number is associated with the sequence length of the miRNA molecule may have some implication in genetic research and is worthwhile to be studied. We retrieved all of the predicted targets from a web tool miRDB [42], [43]. Four mature miRNAs (hsa-miR-3124-5p, hsa-miR-3647-3p, hsa-miR-3647-5p, hsa-miR-3648) were excluded from the analysis because each of them has no predicted target gene according to this tool. Many miRNAs have the same sequence length. Each of these miRNAs binds to at least one gene. **Figure 9** presents the average numbers of predicted target genes versus the mature miRNA sequence length. Generally speaking, the plot shows that the average number of the target genes is positively correlated to the sequence length of the miRNA, and this relationship is statistically significant when considering only the miRNAs with sequence lengths between 17 and 25 (the regression line (red) has a positive slope, 18, with the p value being 0.003),indicating that longer miRNAs tend to regulate more genes. We comment that there are only 17 miRNAs with sequence length of 16, 26 or 27. Nevertheless, this positive correlation is an interesting observation whose biological significance warrants further investigation. Increased miRNA length raises the possibility that the extra nucleotides are involved in facilitating protein complex formation. This could take the form of aiding more stable interactions with core RISC complexes or through interactions with additional regulatory co-factors. An interesting model for the latter is that these additional factors regulate targeting to different subsets of 3′ UTRs depending on the cell phenotype. In this scenario, a broader target repertoire can exist but tissue specific co-factors dictate the subset of targets in a particular tissue.

### The Relationship between miRNA Sequence Length and the Distribution of Target Binding Sites

**Figure 10:**
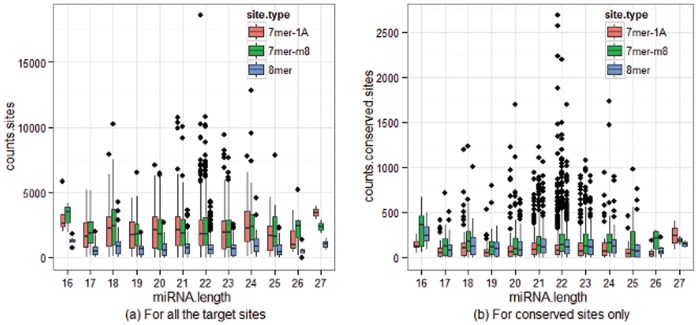
Histograms of target site counts of the transcripts predicted by miRNAs with the same sequence lengths.

In this subsection, we look into the relationship between the lengths and the seed sequences of miRNAs. The motivation for this analysis is that the miRNA target prediction heavily depends on the binding between the seed sequence of the miRNA and the 3′ UTR sequences of the targeted mRNAs [44]. Meanwhile, the non-seed sequence may play some roles in processing of precursor and/or the association with RICS proteins. We downloaded the summary counts, including the numbers of (non-)conserved 8mer sites, 7mer-m8 sites and 7mer-1A sites in the targeted transcripts, from a public domain TargetScan Human Release 6.1 [45]–[48]. This database contains 1524 mature miRNAs. As specified by the website, 8mer refers to a perfect match to nucleotides 2–8 of the mature miRNA (the seed+nucleotide 8) and an A residue at the first nucleotide position of the mature miRNA; 7mer-m8 refers to a perfect match to nucleotides 2–8; and 7mer-1A refers to a perfect match to nucleotides 2–7 plus an A residue at the first nucleotide position. If multiple genes are targeted by a miRNA, we calculate the sum of site counts from each gene and used it as the count of target sites for this miRNA. By doing so, we obtained six numbers, corresponding to (non-)conserved 8mer, 7mer-m8 and 7mer-1A sites, for each miRNA. The distributions of these count numbers are presented in **Figure 10**. The left panel shows the combined counts of conserved and non-conserved sites and the right panel shows the counts of conserved sites only. Both panels indicate the same pattern of count distributions. The distributions of target site counts are very similar for miRNAs with sequence lengths from 17 to 26 (more alike across the sequence lengths from 21 to 23), while the distribution of counts from miRNAs with the shortest or longest sequence shows some discrepancy. Interestingly, for combined counts of conserved and non-conserved sites, the counts of 8-mer sites are much lower than those of 7mer-m8 sites and 7mer-1A sites which have the same medians. This phenomenon changes when only the conserved sites are considered. The median counts of 7mer-1A sites are lowest for miRNAs with sequence lengths from 16 to 26, but highest for miRNAs with sequence length 27.

### Conclusion

The length has become one of the important criteria to annotate a clone sequence as a miRNA [7]. Though it is a common understanding that a human mature miRNA has about 22 nucleotides, the statistical characteristics of the length distribution of the miRNA molecules are not trivial, and have been less studied. Based on graphics methods and the model selection criteria, we demonstrated that, compared with conventionally used Poisson distribution, the discrete analogue of a asymmetric Laplace distribution can nicely model the length distribution of human mature miRNA molecules. It has lower residual sum of squares and smaller AIC. The association study revealed that the sequence length heterogeneity is related to some biological factors such as evolution conservation, miRNA’s regulatory mechanism, etc. We found that highly conserved miRNA sequences are of lengths concentrated at 22 nucleotides while human-specific miRNAs show large variation in the length. Furthermore, the miRNAs that regulate oncogenes/tumor suppressors also show stable lengths of 22 nucleotides, and longer miRNAs tend to regulate more genes. These findings may have some implications on (cancer) genetics research and warrant additional follow-up studies.
